# What is important for doctor’s drug decision-making for the patient with acute schizophrenia?

**DOI:** 10.1192/j.eurpsy.2021.1391

**Published:** 2021-08-13

**Authors:** M. Morozova, G. Rupchev

**Affiliations:** Laboratory Of Psychopharmacology, FSBSI Mental Health Research Center, Moscow, Russian Federation

**Keywords:** Antipsychotic treatment, acute schizophrenia, drug decision-making, clinical symptoms

## Abstract

**Introduction:**

In spite of the long history of antipsychotic treatment there are still no clear criteria, which can be robust support for drug decision-making.

**Objectives:**

To determine the important hallmarks of patient’s current state, life span and history of illness defining the doctor’s decision about the type of antipsychotic to be chosen.

**Methods:**

The data from the case charts of 275 patients with episodic schizophrenia and rather benign course of the disease were analyzed.

**Results:**

The group included: male 62%, mean age 33 (SD 11), education 10 years 23%, 13 years 27%, 16 years 29%, disability - 51%, number of hospitalizations due to psychotic episodes in the past 7 (SD 6). The symptoms of the current episode varied from patient to patient: delusions and hallucinations, symptoms of disorganization, negative symptoms of different severity were registered Atypical antipsychotics were more often than typical prescribed to the patients with developmental problems: traumatic obstetric complications (p=0,009), poor somatic health in the childhood (p= 0,02), cognitive dysfunction during school years (p=0,04), and quality of first remission – presence of residual symptoms in the first remission (p=0.005). Good compliance in the past was one more important factor for choosing a atypical antipsychotic for a patient (p=0.05). It appeared that the most important for the decision-making was the specific features of the patient’s development and early period of the disease, but not the specific signs of current psychotic state.

**Conclusions:**

Doctor’s decision upon the type of antipsychotics in this category of patients is most probably based on other than current clinical symptoms signs.

